# Comparative study on the effect of precursors on the morphology and electronic properties of CdS nanoparticles

**DOI:** 10.3906/kim-2009-9

**Published:** 2021-04-28

**Authors:** Kevin I. Y. KETCHEMEN, Sixberth MLOWE, Linda D. NYAMEN, Peter T. NDIFON, Neerish REVAPRASADU

**Affiliations:** 1 Department of Chemistry, Faculty of Science and Agriculture, University of Zululand, KwaDlangezwa South Africa; 2 Department of Inorganic Chemistry, Faculty of Science, University of Yaoundé I, Yaoundé Cameroon; 3 Department of Chemistry, Dar es Salaam University College of Education, University of Dar es Salaam, Dar es Salaam Tanzania

**Keywords:** Cadmium complexes, dithiocarbamate, xanthate, single source precursors, CdS nanoparticles, optical properties

## Abstract

Cadmium dithiocarbamate and cadmium ethyl xanthate complexes were synthesized and characterized by microanalysis, Fourier transform infrared (FT-IR) spectroscopy and thermogravimetric analyses. The complexes were employed as molecular precursors for the fabrication of CdS nanoparticles in hexadecylamine (HDA) and oleylamine (OLA) at a temperature of 250 °C. Spherical and oval shaped particles with sizes ranging from 9.93 ± 1.89 to 16.74 ± 2.78 nm were obtained in OLA while spherical, oval and rod shaped particles with sizes ranging from 9.40 ± 1.65 to 29.90 ± 5.32 nm were obtained in HDA. Optical properties of the nanoparticles showed blue shifts as compared to the bulk CdS, with the OLA capped nanoparticles slightly more blue shifted than the corresponding HDA capped nanoparticles. Results of crystallinity patterns revealed hexagonal phase of CdS.

## 1. Introduction

II-VI semiconducting nanomaterials have attracted a lot of attention owing to their potential applications in light emitting diodes [1–3], solar cells [4] and optical devices [5]. Cadmium sulfide (CdS) is one of the most studied semiconductors with a direct band gap of 2.42 eV. The thermolysis of complexes as molecular precursors in hot solvents makes it one of the main route to prepare high quality CdS nanocrystals. This synthetic route allows for the monitoring of morphologies of the nanocrystals by varying the temperature of the reaction, the time of the reaction, the concentration of the complex and the capping agent [6,7]. The utilization of molecular precursors produces high quality nanocrystals [8,9].

Complexes of ligands containing sulfur such as dithiocarbamates [10,11], xanthates [12], thiosemicarbazides [13], thiourea [14], dithiophosphinates [15] and dithioimidodiphosphinates [16], have been used as precursors for the synthesis of various metal sulfide nanocrystals of various morphologies ranging from spheres, rods, bipods and tetrapods. Dithiocarbamate and xanthate anions react quickly with metal salts to produce the corresponding metal complexes. Cadmium dithiocarbamate and cadmium xanthate complexes have been used extensively as molecular precursors for the fabrication of CdS nanoparticles of various morphologies ranging from spheres, rods, bipods to tetrapods [11,12,17–20]. Originally, phosphine-based coordinating agents such as tri-n-octylphosphine oxide were employed for surface passivation, however recently alkyl amine coordinating solvents, such as hexadecylamine (HDA) and oleylamine (OLA) have become popular because they can be efficiently bonded to the CdS nanoparticles core to control shape manipulation [21,22].

The success of these metal complexes in the synthesis of CdS particles with interesting shapes has been motivational to researchers in furthering investigations on the influence of the precursor type. We have recently reported the deposition of hexagonal CdS thin films of various morphologies by the AACVD method using Cd(II) dihexyl, diethyl, piperidinyl dithiocarbamates and ethyl xanthate complexes as single source precursors [23]. Herein, we report the preparation of CdS nanocrystals by the thermolysis of Cd(II) dithiocarbamate and ethyl xanthate complexes as single source precursors in oleylamine and hexadecylamine capping solvents.

## 2. Materials and methods

### 2.1. Chemicals

Dihexylamine 97%, hexadecylamine (HDA), hexane, oleylamine (OLA), piperidine 99%, potassium ethyl xanthogenate 96%, trioctylphosphine (TOP) 90%, sodium diethyl dithiocarbamate trihydrate salt of sodium (Sigma–Aldrich Chemie GmbH, Hamburg, Germany), petroleum ether, acetone, cadmium chloride monohydrate 99%, carbon disulfide 99.5%, sodium hydroxide 98%, chloroform, ethanol 99.5% and tetrahydrofuran (THF) (Merck, KGaA, Darmstadt, Germany.) were employed as received without any further purification. The organic ligands and their corresponding Cd(II) complexes were prepared according to the method reported in our very recent work [23] and are detailed in the supplementary document.

### 2.2. Instrumentation

Microanalysis was performed on a Perkin-Elmer automated model 2400 series II CHNS/O analyzer (PerkinElmer Inc., Waltham, MA, USA). Infrared spectra were recorded on a Bruker FT-IR tensor 27 spectrophotometer directly on small samples of the compounds in the range 200–4000 cm^-1^. Thermogravimetric analysis was carried out at 20 °C min^-1^ heating rate using a Perkin Elmer Pyris 6 TGA up to 600 °C in a closed perforated aluminium pan under N2 gas flow. The morphology and particle sizes of the samples were characterised by a JEOL 1010 TEM with an accelerating voltage of 100 kV, Megaview III camera and Soft Imaging Systems iTEM software. A Varian Cary 50 UV-Vis spectrophotometer was used for optical measurements, using silica cuvettes (1 cm path length), and ethanol as a reference solvent at room temperature. Powder X-ray diffraction (p-XRD) were performed on Bruker AXS D8 diffractometer in the angle 2θ range of 20–65 °, equipped with nickel filtered Cu-Kα radiation (λ = 1.54178 Å) at 40 kV, 40 mA, room temperature. 

### 2.3. Synthesis of CdS nanoparticles in HDA and OLA

1.2 mmol of the [Cd(dihex-dtc)2] precursor was dissolved in 6.0 mL of tri-n-octylphosphine (TOP). The resultant mixture was inserted into 6.0 g of hot capping agent (HDA or OLA) in a three-necked flask at 250 ºC. The colour of the solution changed to yellowish-orange with a drop in temperature from 20–30 ºC and the reaction was stabilised at 250 ºC. After an hour, aliquots of the reaction mixture were taken and ethanol added to each, resulting in the appearance of a flocculent solid. Centrifugation of the resulting solution separated out the obtained solid, which was then dispersed in hexane to produce yellowish orange HDA or OLA-capped CdS nanocrystals. The above procedure was then repeated for [Cd(dieth-dtc)_2_], [Cd(pip-dtc)_2_] and [Cd(eth-xan)_2_] complexes.

## 3. Results and discussion

### 3.1. Single source molecular precursors

The complexes used as single source molecular precursors are air and moisture stable at room temperature, easy to prepare and soluble in common organic solvents such as chloroform and THF. Microanalysis confirmed the synthesis of pure compounds. Dihexyl/piperidine dithiocarbamates and the corresponding cadmium(II) complexes prepared from dihexyl/diethyl/piperidine dithiocarbamates and ethyl xanthate (ESI Figure S1) were obtained in good yields, using a simple reaction route and their protocols are reported in our recent work [23]. The IR spectra of cadmium piperidine dithiocarbamate (c), cadmium dihexyl dithiocarbamate (a), cadmium ethyl xanthate (d) and cadmium diethyl dithiocarbamate (b) showed strong broad bands at about 970–980 cm^-1^ attributed to υ(C–S) and the shift of the band at about 1480–1495 cm^-1^, for the different complexes from that of the free ligand and attributed to √(C=N), is indicative of a bidentate complexing [24,25]. The IR data are summarised in the supplementary document (ESI Table S). The TGA profiles of the complexes are shown in ESI Figure S2. All the complexes displayed single step decomposition patterns. 

### 3.2. Synthesis of CdS nanoparticles

Primary amines have been widely employed as capping groups in the synthesis of CdS nanostructured materials since long alkyl chain amines are proving to be appropriate surfactants for semiconducting nanoparticles [10,17–19,26]. The capacity to control the physical and the chemical properties of semiconducting nanoparticles depends on fine-tuning their morphology. The morphology dependent optical properties of such nanostructured materials (spherical, cubic, rod shaped) have been proven to be straight dependent on the resulting shape in the case of semiconductor colloidal nanoparticles [27]. The growth of semiconductor nanoparticles can therefore be observed by the progressive evolution of the UV-visible absorption curve. This enables an understanding of the relationship between reaction parameters and the resultant morphology [10, 11]. In this section, we investigate the effect of the precursor type on the particle size, shape and electronic properties of CdS nanocrystals prepared in hexadecylamine (HDA) and oleylamine (OLA) as coordinating solvents.

#### 3.2.1. TEM studies of CdS nanocrystals

**Figure 1:**
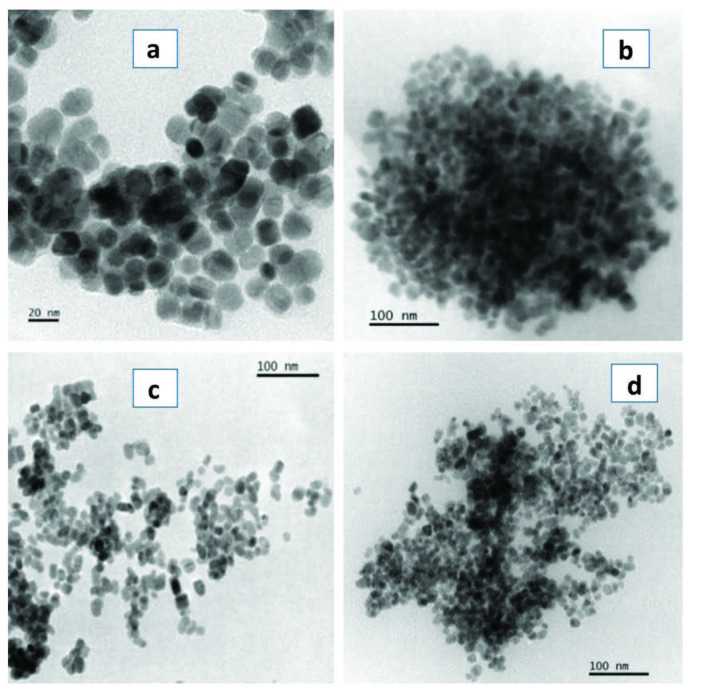
OLA-capped CdS nanoparticles synthesized from a) Cd(II) dihexyl dtc, b) Cd(II) diethyl dtc, c) Cd(II) piperidine dtc and d) Cd(II) ethyl xanthate complexes.

**Figure 2:**
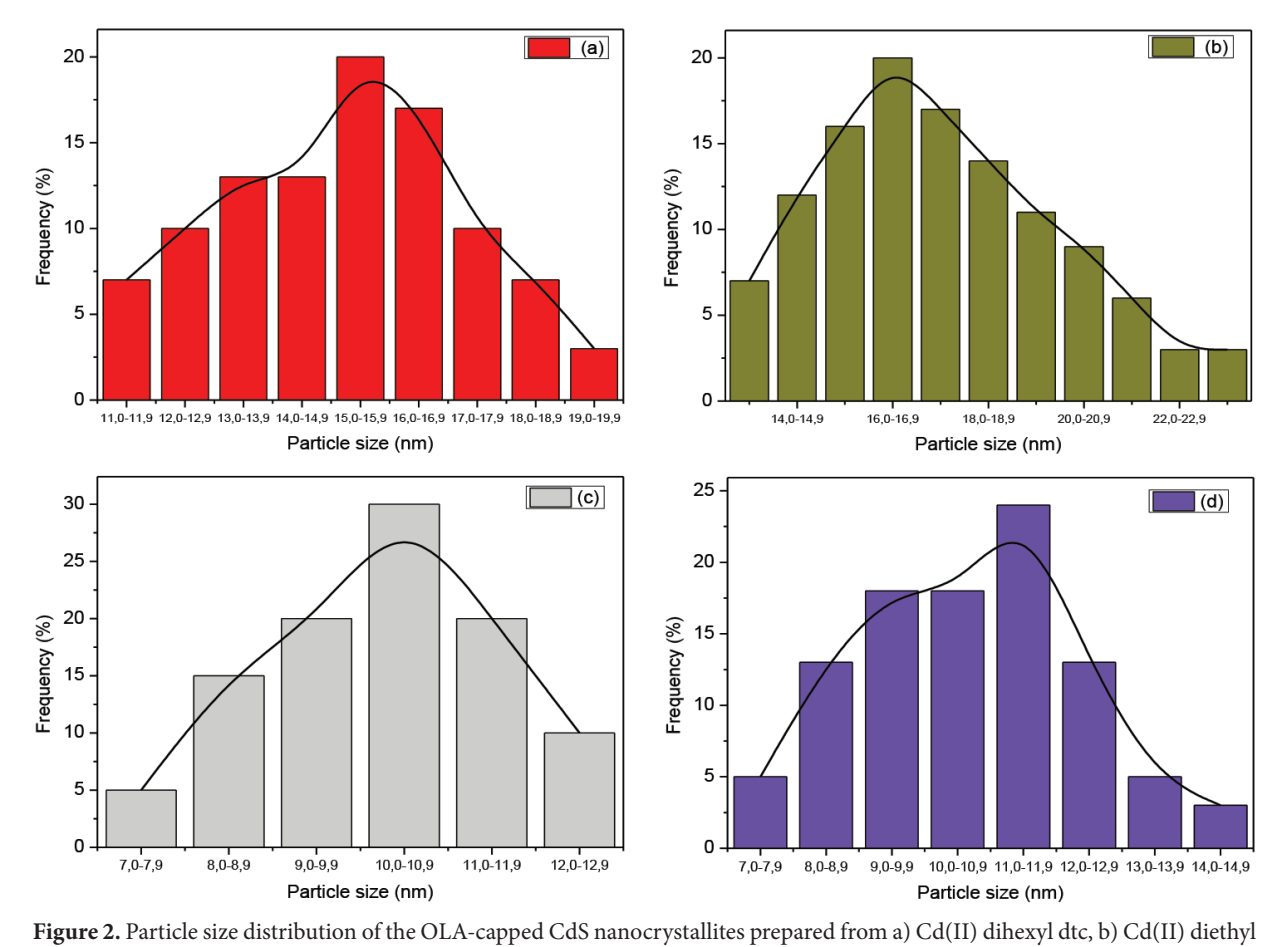
Particle size distribution of the OLA-capped CdS nanocrystallites prepared from a) Cd(II) dihexyl dtc, b) Cd(II) diethyl dtc, c) Cd(II) piperidine dtc and d) Cd(II) ethyl xanthate complexes.

The favoured growth regime of the reaction is influenced by the ultimate morphology of nanocrystals. The reaction can go on in either a kinetic or a thermodynamic growth regime [28,29]. The TEM images of the OLA capped CdS nanoparticles (Figure 1) showed that, spherical to cubic shaped particles were obtained from the Cd(II) dihexyl dtc complex with an average particle size of 13.91 ± 2.68 nm. Oval shaped particles with an average size of 16.74 ± 2.78 nm were obtained from the Cd(II) diethyl dtc complex. When the precursor was changed from the alkyl chain to a heterocyclic one, oval shaped particles with an average size of 9.93 ± 1.89 nm were obtained from the Cd(II) piperidine dtc complex. When the precursor was changed to a xanthate complex, spherical to oval shaped particles with an average size of 9.48 ± 1.75 nm were obtained from the Cd(II) ethyl xanthate and their corresponding particles size distributions are presented in (Figure 2). 

**Figure 3:**
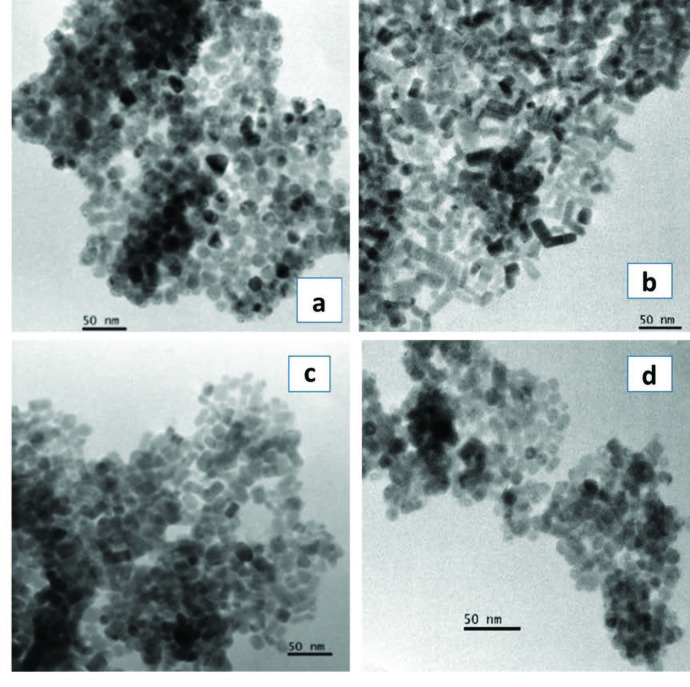
HDA-capped CdS nanoparticles prepared from a) Cd(II) dihexyl dtc, b) Cd(II) diethyl dtc, c) Cd(II) piperidine dtc and d) Cd(II) ethyl xanthate complexes.

**Figure 4:**
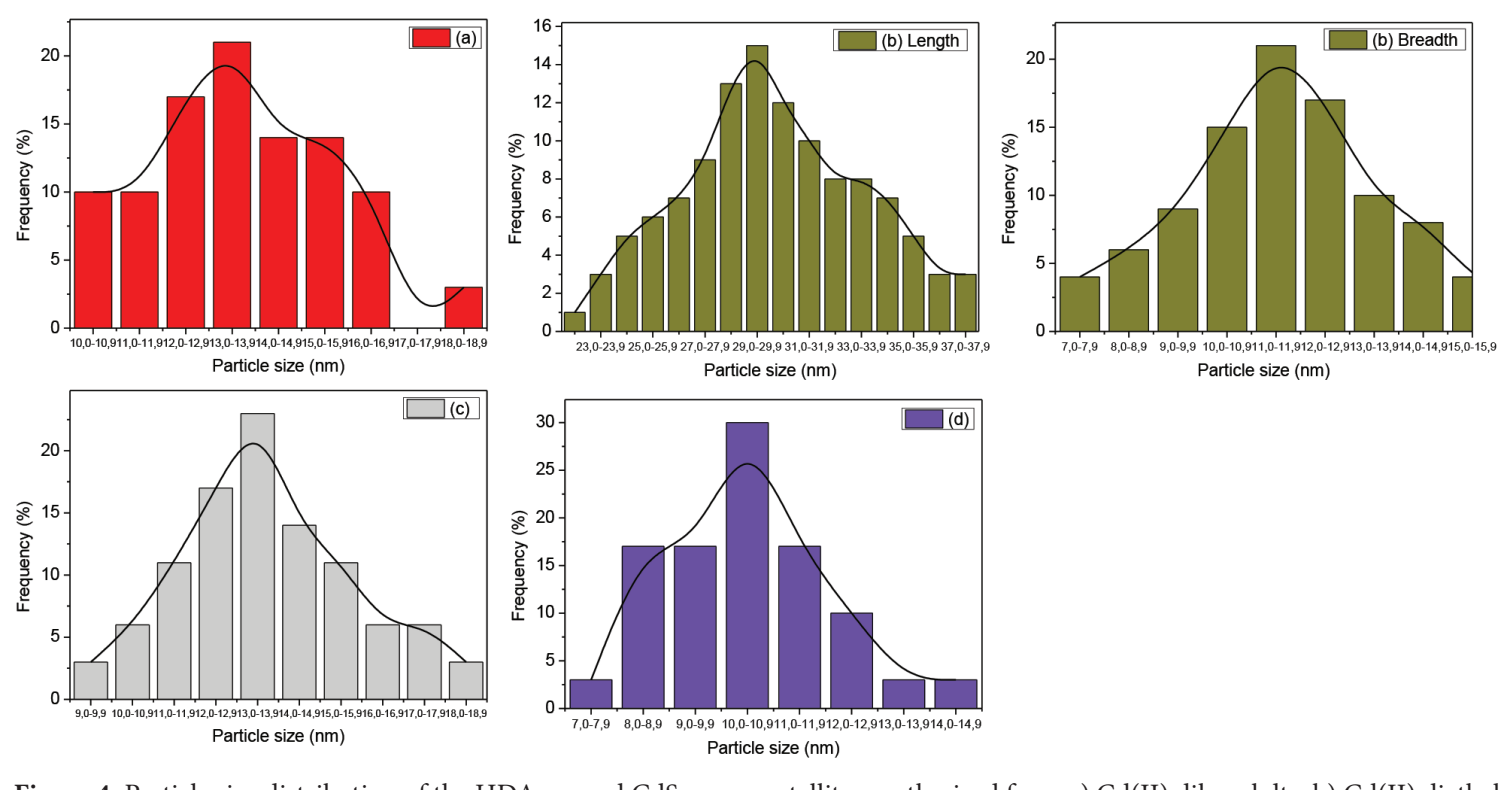
Particle size distribution of the HDA-capped CdS nanocrystallites synthesized from a) Cd(II) dihexyl dtc, b) Cd(II) diethyl dtc, c) Cd(II) piperidine dtc and d) Cd(II) ethyl xanthate complexes.

On the other hand, the TEM images of HDA capped CdS nanoparticles (Figure 3) showed that, spherical shaped particles with a mean diameter of 12.93 ± 2.45 nm were obtained from the Cd(II) dihexyl dtc complex and rod-shaped nanoparticles with a mean length of 29.90 ± 5.32 nm and a breadth of 11.31 ± 2.37 nm were also obtained from the Cd(II) diethyl dtc complex with an aspect ratio of 2.64. When the precursor was changed from alkyl to heterocyclic, oval to rod shaped particles with an average particle size of 13.63 ± 2.68 nm were obtained from the Cd(II) piperidine dtc complex. On the other hand, the Cd(II) ethyl xanthate complex gave spherical to oval shaped particles with a mean diameter of 9.40 ± 1.65 nm. Their particles size distributions are presented in (Figure 4).

It is observed that different morphologies and sizes were obtained for each precursor using different capping groups (Table). The shape and size of CdS nanoparticles varied slightly when we used complex (a) and (d) and the variation was more pronounced when we used complex (b) and (c) in the different capping agents. The nanoparticles obtained from the Cd(II) xanthate complex were larger in size than those obtained from the Cd(II) dithiocarbamates complexes. The sizes of the OLA-capped nanoparticles decreased when obtained from Cd(II) diethyl dtc, Cd(II) dihexyl dtc, Cd(II) piperidine dtc and Cd(II) ethylxanthate respectively. When HDA was used as a capping agent, a similar order was observed namely; Cd(II) diethyl dtc, Cd(II) piperidine dtc, Cd(II) dihexyl dtc and Cd(II) ethyl xanthate. This observation can be explained by the influence of the length of the alkyl chain of the capping group in controlling size and shape [11]. The introduction of surfactants that adsorb onto the surfaces of the growing particles can moderate the surface energy of the nanocrystals thereby significantly influencing the morphology of the crystals. Hexadecylamine is reported to dynamically adsorb on the surface of the growing crystal [10]. Oleylamine has a double role in being a strong capping group, and also is identified to aid in the decomposition of the precursor complex. Various reaction parameters such as the precursor type, reaction temperature and capping agent govern if the reaction can take place in a kinetic or thermodynamic regime [30,31]. 

**Table:** Optical properties and particle sizes in nanometer (nm) for CdS nanocrystals prepared using complexes (a), (b), (c) and (d) under various reaction conditions.

Cd(II) complex	Decomposition temperature (°C)	Capping agent	Absorption band edge (nm)	Shape	TEM particle size(nm)	XRD (110) particle size (nm)
(a)	250	OLA	490	Spherical to cubic	13.91 ± 2.68	13.56
(b)			480	Oval	16.74 ± 2.78	13.60
(c)			495	Oval	9.93 ± 1.89	13.14
(d)			485	Spherical to oval	9.48 ± 1.75	11.45
(a)	250	HDA	505	Spherical	12.93 ± 2.45	13.50
(b)			485	Rod	Length: 29.90 ± 5.32Breadth: 11.31 ± 2.37	/
(c)			500	Oval to rod	13.63 ± 2.68	13.00
(d)			490	Spherical to oval	9.40 ± 1.65	11.67

#### 3.2.2. Optical properties of CdS nanoparticles

**Figure 5:**
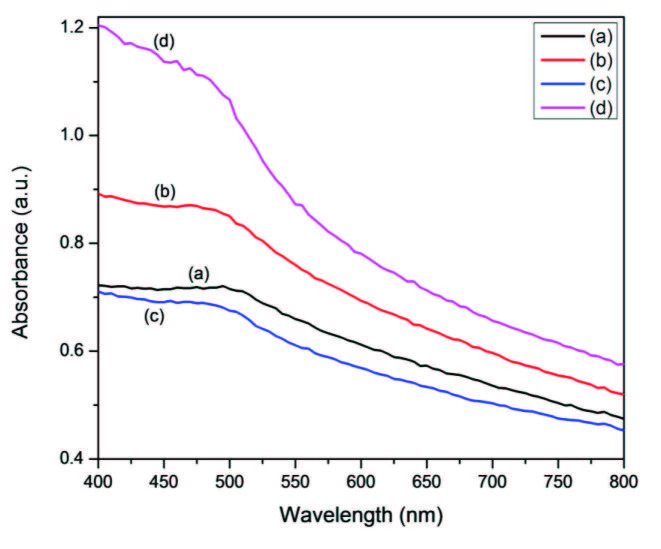
UV-visible of HDA-capped CdS nanoparticles prepared from a) Cd(II) dihexyl dtc, b) Cd(II) diethyl dtc, c) Cd(II) piperidine dtc and d) Cd(II) ethyl xanthate complexes.

**Figure 6:**
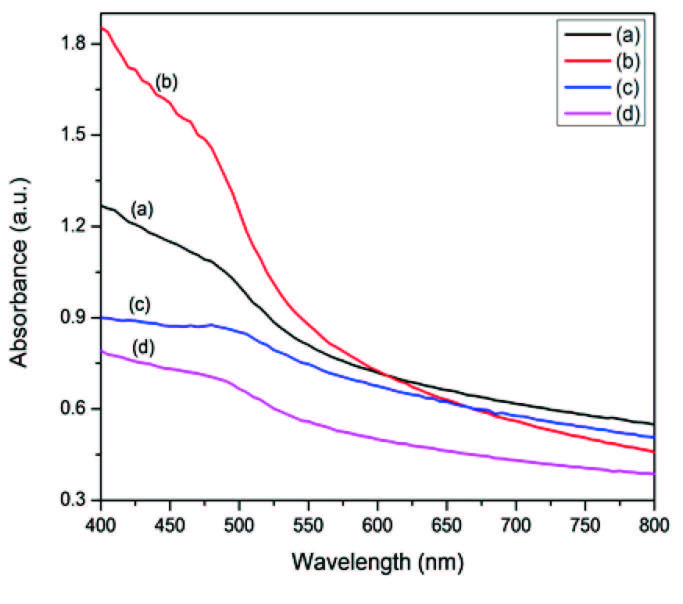
UV-visible of OLA-capped CdS nanoparticles prepared from a) Cd(II) dihexyl dtc, b) Cd(II) diethyl dtc, c) Cd(II) piperidine dtc and d) Cd(II) ethyl xanthate complexes.

The influence of the precursor on the optical properties of CdS nanocrystals was investigated. The absorption spectra of the prepared HDA-capped and OLA-capped CdS nanocrystals are presented in Figures 5 and 6, respectively. Almost all the samples show an absorption shoulder (see the values in Table). The band edge of all the samples falls in the 480–495 nm range when OLA was employed as a capping group. When HDA was used, the band edge is between 485–505 nm with respect to bulk CdS. In the spectra of OLA capped CdS, we noticed a band edge of 495 nm for CdS nanoparticles prepared from the Cd(II) piperidine dtc complex which was more bathochromically shifted while the band edge from Cd(II) diethyl dtc complex of 480 nm was the least shifted. The spectra of HDA capped CdS nanocrystals prepared from Cd(II) dihexyl dtc complex with a band edge of 505 nm was also bathochromically shifted while the one from Cd(II) diethyl dtc complex (485 nm) remained less shifted. The UV-visible absorption and the corresponding TEM images of the CdS nanocrystals presented in Figure S3 reveal that the particles are all blue shifted as compared to the bulk CdS (515 nm). The variation of the precursor type had some effects on the optical properties. It is well known that the nature of the precursor plays a significant role in the final morphology as well as optical properties of the crystals [6]. Therefore, the rate of the reaction is considerably affected by the nature of precursor decomposition. The OLA capped CdS nanoparticles are more blue shifted than the corresponding HDA capped CdS nanoparticles (Figure S4). This could be explained by the fact that the higher reaction temperature results in the formation of irregular nanocrystals with reduced crystallinity due to random and faster nucleation [32]. 

#### 3.2.3. Powder-XRD studies of CdS nanocrystals

**Figure 7:**
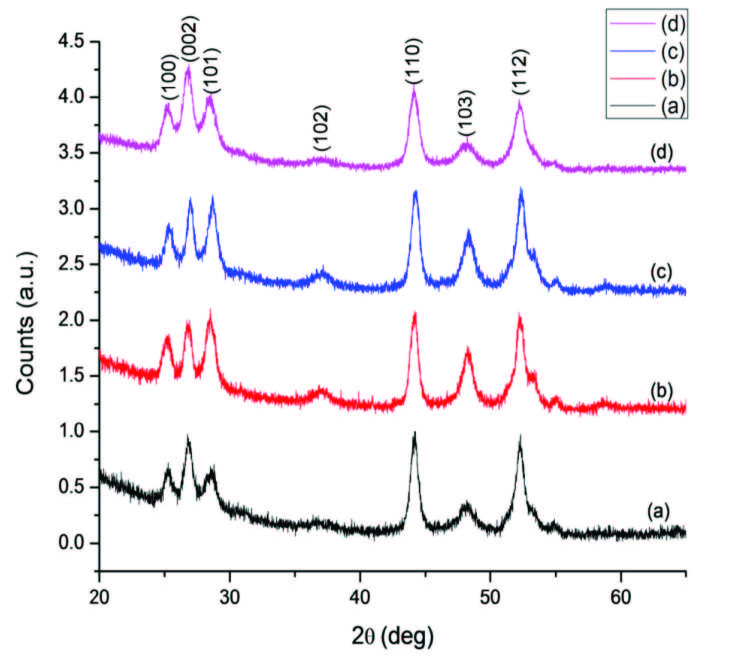
P-XRD patterns of OLA-capped CdS nanocrystals prepared from a) Cd(II) dihexyl dtc, b) Cd(II) diethyl dtc, c) Cd(II) piperidine dtc and d) Cd(II) ethyl xanthate complexes.

The crystal phase of the CdS nanocrystals obtained from all the precursors was examined by X-ray diffraction (XRD), as presented in Figures 7 and S5. The XRD reflections planes were indexed and all are almost identical with the CdS wurtzite crystalline system (card number: 01-077-2306). Diffraction peaks at 2θ = 24.84º, 26.53º, 28.22º, 43.74º, 47.89º and 51.89º corresponding to the (100), (002), (101), (110), (103) and (112) planes of wurtzite CdS respectively were found in both diffraction patterns. The broadness of the p-XRD diffraction peaks was indicative of the relatively small particle size and heterogeneous particle size distribution [33]. The higher intensity (002) peak observed in the diffraction pattern indicated preference elongation along the c-axis [34]. Yet, the (102) plane confirmed the dominance of the hexagonal phase and a nonindexed peak at 2θ = 23.27º which was observed in addition when HDA was used, probably due to the presence of trace amounts of precursor complexes. Furthermore, we noticed that the crystal phase of CdS nanomaterials was not influenced by the difference in chemical composition for each single-source precursor.

## 4. Conclusion

We have successfully prepared cadmium(II) dihexyl, diethyl, piperidine dithiocarbamates and cadmium(II) ethyl xanthate complexes and employed them as molecular precursors for the preparation of CdS nanocrystals by hot injection in OLA and HDA as capping agents at 250 °C. When thermolysis was carried out in OLA, spherical-oval to cubic shaped nanoparticles were obtained for CdS nanocrystallites whereas in HDA, spherical, oval and rod shaped CdS nanoparticles were obtained. The mean particle sizes were calculated by TEM measurements, which revealed that particle sizes and morphologies varied depending on the ligand chain. Optical absorption values show a blue shift as compared to the bulk CdS and were thus consistent with the literature values. Moreover, the p-X-ray diffraction results showed a single hexagonal phase for all CdS nanoparticles.
